# The Interplay between Non-Esterified Fatty Acids and Plasma Zinc and Its Influence on Thrombotic Risk in Obesity and Type 2 Diabetes

**DOI:** 10.3390/ijms221810140

**Published:** 2021-09-20

**Authors:** Stephen J. Hierons, Jordan S. Marsh, Dongmei Wu, Claudia A. Blindauer, Alan J. Stewart

**Affiliations:** 1 School of Medicine, University of St. Andrews, St. Andrews KY16 9TF, Fife, UK; sh256@st-andrews.ac.uk (S.J.H.); jm451@st-andrews.ac.uk (J.S.M.); dw95@st-andrews.ac.uk (D.W.); 2 Department of Chemistry, University of Warwick, Coventry CV4 7AL, UK; c.blindauer@warwick.ac.uk

**Keywords:** diabetes, human serum albumin, insulin resistance, non-esterified fatty acids, obesity, thrombosis, zinc

## Abstract

Thrombosis is a major comorbidity of obesity and type-2 diabetes mellitus (T2DM). Despite the development of numerous effective treatments and preventative strategies to address thrombotic disease in such individuals, the incidence of thrombotic complications remains high. This suggests that not all the pathophysiological mechanisms underlying these events have been identified or targeted. Non-esterified fatty acids (NEFAs) are increasingly regarded as a nexus between obesity, insulin resistance, and vascular disease. Notably, plasma NEFA levels are consistently elevated in obesity and T2DM and may impact hemostasis in several ways. A potentially unrecognized route of NEFA-mediated thrombotic activity is their ability to disturb Zn^2+^ speciation in the plasma. Zn^2+^ is a potent regulator of coagulation and its availability in the plasma is monitored carefully through buffering by human serum albumin (HSA). The binding of long-chain NEFAs such as palmitate and stearate, however, trigger a conformational change in HSA that reduces its ability to bind Zn^2+^, thus increasing the ion’s availability to bind and activate coagulation proteins. NEFA-mediated perturbation of HSA-Zn^2+^ binding is thus predicted to contribute to the prothrombotic milieu in obesity and T2DM, representing a novel targetable disease mechanism in these disorders.

## 1. Introduction

Obesity and type-2 diabetes mellitus (T2DM) are two closely related disorders, the former being associated with a high body mass index (BMI; >30 kg/m^2^) and the latter with insulin resistance and inadequate glycemic control. Both conditions predispose an individual to vascular complications [1,2,3,4]. Increased vascular risk in these diseases is driven by the establishment of a hypercoagulable state of the plasma [5], which can include the formation of intravascular obstructive blood clots (leading to heart attacks and strokes). Such thrombi are formed secondarily to complex interactions between platelets and coagulation proteins (comprising the cellular and protein arms of coagulation, respectively). Both these aspects of coagulation are altered in obesity and T2DM; platelets display hyperactivity, while coagulation proteins circulate at higher concentrations and display enhanced activation, ultimately leading to the formation of compact fibrin networks and impaired fibrinolysis [5,6]. Hypofibrinolysis also represents a key abnormality in obese and diabetic patients and contributes to the adverse clinical outcome in this population [7,8].

In addition to an enhanced thrombotic state, obesity and T2DM are also associated with derangements in lipid metabolism with resulting changes to the plasma lipid profile of affected individuals [9,10]. Of particular interest is the impact of these disease states on the levels of plasma non-esterified fatty acids (NEFAs). As a major reservoir of metabolic energy, one of the primary components of cellular membranes and a precursor to numerous cellular effectors, plasma NEFA levels can be altered in a variety of disease states. Notably, plasma NEFA levels are frequently elevated in obesity and T2DM [11,12,13,14,15], likely resulting from imbalances between uptake and release of NEFAs by adipocytes. In particular, basal fat cell lipolysis (the hydrolysis of triacylglycerol into fatty acids and glycerol) is elevated in the obese state, resulting in greater release of NEFAs into the blood [16]. Enlarged and metabolically stressed adipose tissue also loses the ability to adequately store fat over time, resulting in greater migration of NEFAs towards ectopic tissues such as the liver and skeletal muscle [17]. Elevations in plasma NEFA levels have relevant pathological implications. Total NEFA concentration is closely related to insulin resistance, and NEFAs may have a causative role in its onset [12,18,19]. Importantly, adipose tissue lipolysis is inhibited by insulin [20], and so this mechanism to control NEFA release is also lost in the diabetic state, likely causing further elevations in plasma NEFA levels.

Plasma NEFA levels are related to unfavorable prognoses in both stroke and cardiovascular disease [21,22,23] and are likely to contribute to the prothrombotic milieu in obesity and T2DM. Several mechanisms by which NEFAs contribute to thrombosis have already been proposed. These include their ability to induce endothelial dysfunction (and, thus, contribute to atherosclerosis) [24], to alter platelet function [25], and to directly dysregulate fibrin clot structure [26]. Additionally, recent findings also highlight the role of NEFAs as indirect effectors of coagulation through their ability to impact zinc availability in the plasma. Zinc is an essential regulator of coagulation, mediating multiple aspects including platelet aggregation, fibrin clotting, and fibrinolysis [27]. Specifically, these effects are mediated by the free aquo ion of Zn^2+^. Extracellular Zn^2+^ is bound by human serum albumin (HSA), which serves as the major buffering agent of plasma Zn^2+^. HSA also binds and transports NEFAs at several sites [28,29,30] and, importantly, pathophysiological concentrations of these fatty acids (as documented in obesity and T2DM) perturb the ability of HSA to bind and buffer Zn^2+^ [31]. As a consequence of this allostery, Zn^2+^ no longer able to bind HSA has greater freedom to bind and activate clot-forming proteins. It is predicted that sustained prothrombotic signaling by this Zn^2+^ fraction contributes to the greater clotting risk observed in obesity and T2DM [32].

## 2. Zn^2+^ as a Regulator of Hemostasis

**Figure 1:**
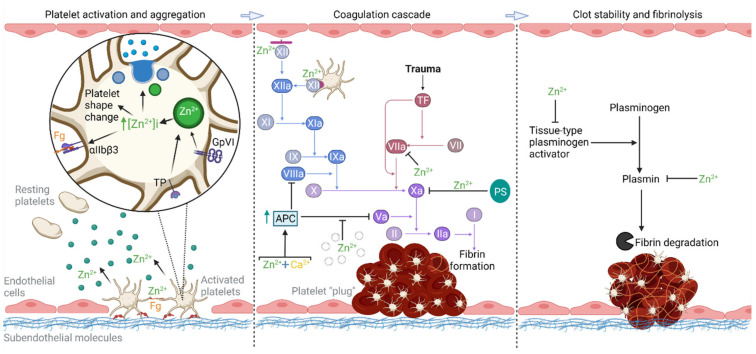
The role of Zn^2+^ in hemostasis. Zn^2+^ is an important mediator of hemostasis, affecting several key aspects of the process. Platelet activation and aggregation: Platelets bind to exposed subendothelial molecules and deposited von Willebrand factor. When collagen and thromboxane A2 bind to platelet receptors, glycoprotein (GP) VI and thromboxane receptor (TP), respectively, it is likely that a cytosolic increase in Zn^2+^ results, from α-granules, leading to platelet shape change, granule release, and activation of αIIbβ3. Platelets bind fibrinogen (fg) through their activated αIIbβ3 integrin to aggregate, a process enhanced by Zn^2+^. Granule release causes increases in extracellular Zn^2+^, potentiating and activating nearby platelets. Coagulation cascade: Zn^2+^ can directly or indirectly modulate the activity of several coagulation factors. Pro-coagulant effects by Zn^2+^ include the facilitation of the intrinsic pathway through enabling FXII binding to platelet and endothelial surfaces and, in the presence of phospholipid vesicles, attenuating the inhibitory action of activated protein C (APC) on FVa. Anti-coagulant effects by Zn^2+^ include the direct inhibition of FXa production through FVIIa binding, increased binding of protein S to FXa (attenuating its activity) and, in the presence of Ca^2+^, promotion of APC generation. The coagulation cascade is highlighted in blue, red and purple to illustrate the intrinsic, extrinsic and common pathways respectively. Clot stability and fibrinolysis: Zn^2+^ promotes thrombus longevity by attenuating components of the fibrinolytic system. Created with BioRender.com (accessed 27 August 2021).

Thrombus formation is the result of hemostasis, a normal physiological process that maintains blood vessel integrity and responds to and blocks vascular breach. The hemostatic mechanism occurs through the actions of both cellular and soluble protein components, namely, platelets and clotting factors, respectively. Platelet activation and aggregation result in the formation of an initial platelet “plug” at the site of vascular damage. In turn, protein clotting factors undergo sequential activation (through the coagulation cascade), culminating in the generation of a fibrin network that stabilizes the platelet aggregate [33]. These two systems (termed primary and secondary hemostasis, respectively) are not separate but instead operate simultaneously, synergistically, and in a linked manner [34,35]. Following early observations that zinc deficiency was associated with bleeding and clotting impairments, research has since identified the Zn^2+^ ion as a ubiquitous agent in the coagulation mechanism [27]. Importantly, free Zn^2+^ has a role in controlling both platelet function and fibrin network formation both directly and indirectly (Figure 1).

The initial response to vascular damage is the attachment and activation of platelets at the site of injury. Activated platelets subsequently aggregate together to form an occluding platelet “plug” (primary hemostasis) to temporarily stop bleeding [35]. Platelets can facilitate this process, along with the generation of fibrin (secondary hemostasis), through the release of numerous pro-coagulant factors that originate from internal stores known as dense and α-granules [35,36]. This latter granule type is the most abundant and is a major store of Zn^2+^ within platelets (containing 40% of total platelet zinc content) [36,37,38]. Trauma to the vasculature and subsequent loss of endothelial cells results in the presentation of subendothelial molecules (such as collagen, fibronectin, and laminin [39,40,41]). This creates anchorage sites, further enhanced by the deposition of plasma von Willebrand factor onto collagen, for platelets to bind [40,42]. Platelet adhesion activates platelets and begins a myriad of processes such as morphological changes and platelet aggregation (through activation of αIIbβ3 integrins [43,44]). Elevated cytosolic Zn^2+^, which can occur following agonist binding to glycoprotein (GP) VI (a platelet receptor for collagen), has been shown to promote these downstream effects along with dense granule release [45]. Consequently, Zn^2+^ has been proposed as a secondary messenger within platelets [45]. Interestingly, cytosolic Zn^2+^ is also elevated with agonist binding to the thromboxane receptor, a receptor for thromboxane A2 (produced by activated platelets), suggesting that cytosolic Zn^2+^ may be important in both the initial and later stages of platelet activation [45]. Activated platelets mobilize their granules, which fuse with the plasma membrane and the open canicular system (a unique membrane system made up of invaginations) to release their contents into the extracellular space [46]. Transient elevations of free Zn^2+^ in the microenvironment around a site of injury are driven by the release of Zn^2+^ from both activated platelets and nearby damaged cells [38,47]. This free Zn^2+^ can be taken up by nearby resting platelets and, depending on the concentration, can either directly activate platelets or potentiate the response of platelets to other platelet agonists [48]. Circulating fibrinogen can also be bound by neighboring platelets, through the now active αIIbβ3 integrin, linking them together and facilitating thrombus formation and growth [49]. Zn^2+^, released from platelets, further promotes aggregation by amplifying both the number of fibrinogen binding sites and agonist-induced platelet aggregation [50,51,52].

During secondary hemostasis, the platelet plug (the final product of primary hemostasis) is stabilized using an interlaced fibrin mesh, which is itself the final product of the coagulation cascade. The coagulation cascade is initiated through two pathways, intrinsic and extrinsic, which merge into a common pathway to facilitate a thrombin spike and, consequently, fibrinogen to fibrin conversion [35]. The intrinsic pathway starts with activation of factor (F) XII, which can occur through autoactivation on negatively charged molecules and surfaces [53]. This includes artificial surfaces, activated platelets, or endothelial cells (with Zn^2+^ an essential cofactor for the former two surfaces) [27,53,54,55,56]. Alternatively, Factor XII can be activated through the contact activation system, with Zn^2+^ again a cofactor in the interaction between high-molecular-weight kininogen (HMWK) and FXII with endothelial cells [57,58]. The pathway continues with FXIIa converting FXI to FXIa, which occurs when FXI binds onto the platelet surface (an interaction enhanced by Zn^2+^) [59,60]. FXIa can then activate FIX, allowing the intrinsic tenase complex to be formed, which subsequently activates FX to meet the extrinsic pathway [60]. In the extrinsic pathway, as blood flows out the damaged site, the circulating FVII encounters tissue factor (TF) and forms the activated complex TF-FVIIa, which can then activate FX [60]. Ultimately, the two coagulation pathways converge and result in thrombin generation, enabling the cleavage of fibrinogen to form fibrin, with Zn^2+^ able to bind to both [60,61]. Interestingly, the absence of the intrinsic coagulation pathway (through congenital FXII deficiency) does not prevent hemostasis and has been implicated in thrombosis (where mice deficient in FXII- or FXI, factors of the intrinsic pathway, displayed a protective effect against thrombosis) [62].

Multiple facets of clot stability, generation, and degradation can be affected by the presence of Zn^2+^, which decreases fibrin generation time, reduces fibrin stiffness, and produces thicker fibrin fibers [63,64]. Though this last feature can render a thrombus more susceptible to fibrinolysis, Zn^2+^ can inhibit this process by reducing tissue-type plasminogen activator (tPA)-mediated plasminogen activation, and the activity of plasmin [63,64]. Additionally, promotion of the heparin-thrombin-fibrin complex by Zn^2+^ can shield thrombin from inactivation by antithrombin (since heparin binding to both antithrombin and thrombin is required for the latter’s inhibition) and enable further fibrin generation to occur [65].

Finally, it is noted that the hemostatic contributions of Zn^2+^ are not solely of a procoagulant nature. Indeed, coagulation can also be attenuated by Zn^2+^ through its action on several proteins, altering their affinities and activities. Zn^2+^ promotes the interaction of HMWK and FXII with GPIbα, a subunit of the GPIb-V-IX complex, resulting in reduced thrombin binding to GPIbα [66,67]. Though thrombin (a potent platelet agonist) can activate platelets without binding GPIbα, it is thought that the maximal effect of thrombin requires this interaction [67]. Additionally, Zn^2+^ amplifies the anti-coagulant effects of protein S, inhibits the pro-coagulant FVIIa, and has a varying coagulant effect on activated protein C (depending on other components present; Figure 1) [27]. Overall, however, several notable observations give prominence to the role of Zn^2+^ as an important effector of coagulation. This includes the ability of Zn^2+^ to regulate platelet aggregation, to act as a cofactor in the coagulation cascade, and to directly alter the properties of the platelet-fibrin thrombus. Given these considerations, the availability of Zn^2+^ in plasma must be adequately controlled to prevent improper activation of Zn^2+^-mediated coagulation pathways.

## 3. Control of Plasma Zn^2+^ Availability and the Impact of NEFAs

The task of buffering (and transporting) Zn^2+^ in the blood is overwhelmingly performed by human serum albumin (HSA) [68]. HSA folds into three homologous domains (I, II, and III) and each domain is formed by two subdomains (A and B). HSA is the dominant protein in adult plasma (~40 mg mL^−1^ [69]) and is reported to bind 75–85% (9–14 μM) of the Zn^2+^ circulating in the blood [70]. This makes HSA the major regulator of Zn^2+^ “speciation” in the plasma, where speciation refers to the state in which Zn^2+^ is present (bound or unbound; and, if bound, to which partner molecule(s)). HSA possesses two Zn^2+^-binding sites that have been experimentally identified, namely, site A and B. Site A has been recognized as the primary Zn^2+^-binding site to HSA (with a *K*_D_ of 100 nM), while site B is the secondary site (with a *K*_D_ in the mid-micromolar range) and is unlikely to contribute greatly to Zn^2+^ binding in vivo [68]. Site A is located at the interface between domains I and II and includes His67 (from domain I) and His247 and Asp249 (from domain II) [71,72]. It has been suggested that site B may also be an interdomain site, composed of residues His9, Asp13 (from domain I), and Asp255 (from domain II) by our previously elucidated structures of Zn^2+^-bound human and equine serum albumin [72].

HSA has a highly dynamic structure, and this dynamicity endows the HSA molecule with considerable binding versatility. In the blood, the protein acts as a repository in which a large variety of endogenous and exogenous molecules may be stored and transported [73]. The binding, transport, and release of its cargos are strongly dependent on HSA conformation, which itself is readily influenced by numerous physiological and pathophysiological conditions including pH, endogenous molecules, and post-translational modifications [74]. Notably, in diabetes, the properties of HSA have been shown to change in a manner that impacts its Zn^2+^-binding ability [75]. Poor glycemic control (in both types I and II diabetes) causes a marked increase in glycated HSA levels with concomitant change to both HSA structure and circulatory half-life [76,77,78,79,80]. Using spectrometric and calorimetric approaches, a reduced affinity for Zn^2+^ has been demonstrated in glycated HSA [75], potentially due to local unfolding of the protein (in subdomain IIA, which harbors two of the three Zn^2+^-binding residues). Furthermore, a comparison of 11.5% and 65.5% glycated HSA (which are in the pathophysiological range [81]) revealed the affinity of the latter for Zn^2+^ to be 2.3-fold lower [82].

**Figure 2:**
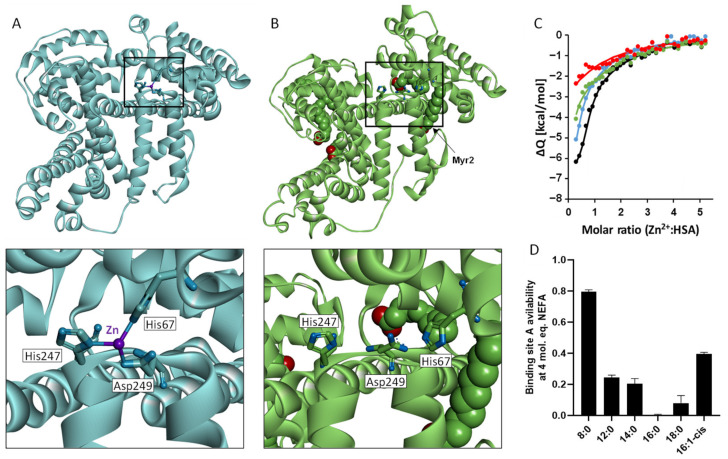
Influence of fatty acids on HSA structure and zinc binding. (**A**). X-ray crystal structure of HSA with zinc bound (PDB: 5IJF). Zinc binds in a tetrahedral geometry at site A involving the side chains of His67, His247, and Asp249. (**B**). X-ray crystal structure of HSA with myristate bound (PDB: 1BJ5). The binding of myristate at the FA2 site causes movement of zinc-binding residue His67 away from His247 and Asp249. (**C**). Isothermal titration calorimetry showing the effect of fatty acid loading on zinc binding to HSA. In the experiments 1.5 mM ZnCl_2_ was titrated into 60 µM HSA, in the presence of either 0 (black), 3 (blue), 4 (green), or 5 (red) mol. eq. of myristate in a buffer containing 50 mM Tris, 140 mM NaCl, pH 7.4. (**D**). Bar chart representing the availability of binding site A in the presence of 4 mol. eq. of various fatty acids. All except octanoate had large effects on Zn^2+^ binding to the protein. Data for parts C and D were taken from Sobczak et al. [88].

In addition to its role as the primary Zn^2+^ carrier, HSA is also the principal transporter of NEFAs in the blood. There are at least seven NEFA-binding sites (FA1-7) on HSA that have been identified by crystallographic studies. These sites are asymmetrically distributed across its three domains, with FA2 (domain I/II interface), FA4, and FA5 (both domain III) considered as the highest-affinity sites [28,29]. Under normal physiological conditions, HSA binds between 0.1–2 molar equivalents (mol. eq.) of NEFAs, depending on metabolic demand. However, HSA is capable of binding much higher NEFA concentrations (up to 6 mol. eq.) in certain pathological conditions, which include T2DM and cardiovascular disease [73]. Multiple studies have demonstrated the ability of NEFAs to influence the ligand-binding properties of HSA [30,83,84,85,86]. Notably, recent evidence has indicated that NEFA binding to the FA2 site prevents Zn^2+^ binding at site A, leading to a considerable reduction in Zn^2+^ binding affinity (as summarized in Figure 2A,B). NEFA binding at the FA2 site requires the interaction of residues from both domains I and II. In particular, the methylene tail of the NEFA molecule makes contract with predominantly hydrophobic residues in sub-domains IA and IB. The carboxylate end, meanwhile, is anchored by residues Arg257 and Ser287 from sub-domain IIA and Tyr150 from sub-domain IIB [29]. In apo-HSA, the two half-pockets in the two domains are ~10 Å apart [32,87]. Accommodation of a NEFA molecule requires a substantial domain–domain movement to bring them together. The resulting conformation change also affects subdomain IA and moves the Zn^2+^-coordinating nitrogen of His67 (domain I) approximately 8 Å away from its initial position in the proximity of His247 and Asp249 (domain II), too far to form a viable Zn^2+^-binding site. Thus, NEFA binding at the high-affinity site FA2 prevents coordination of any previously bound Zn^2+^ ion [31,32]. The ability of different NEFAs (octanoate (C8:0), laurate (C12:0), myristate (C14:0), palmitate (C16:0), palmitoleate (C16:1-cis), and stearate (C18:0)) to influence Zn^2+^ binding to HSA has been examined through competition experiments using isothermal titration calorimetry (Figure 2C,D). Addition of up to 5 mol. eq. of octanoate had little effect on Zn^2+^ binding to HSA (it is too short to elicit the conformational switch), but a change was seen with laurate and longer-chain saturated NEFAs, where the results suggested a reduction in the stoichiometry of site A with increasing NEFA concentrations [88].

## 4. Impact of NEFAs on Zn^2+^–Protein Interactions

NEFAs are clearly capable of reducing the Zn^2+^-binding ability of HSA. Moreover, recent evidence from Coverdale et al. has demonstrated that this allosteric interaction is sufficient to alter the speciation of Zn^2+^ in plasma [31]. Metalloproteomic analysis of fractionated plasma (in the absence and presence of 5 mol. eq. myristate relative to HSA) revealed the concentration of Zn^2+^ in HSA-containing fractions to be reduced in myristate-treated plasma, with a shift to proteins with higher molecular weight. Thus, it is likely that in conditions of obesity and T2DM (where plasma NEFA levels are consistently elevated) Zn^2+^ speciation is chronically disrupted. Altered Zn^2+^ speciation and, hence, dynamics brought about by elevated plasma NEFA may ultimately have the potential to trigger and/or potentiate thrombotic events, thus constituting a novel mechanism of thrombosis in obesity and T2DM. Zn^2+^ ions not able to bind to HSA may drive thrombus formation by acting on fibrin clot formation and lysis directly, for example, by increasing the rate of clot formation (thus enhancing clot stability) or by delaying clot lysis through attenuation of plasmin-mediated fibrin degradation [32]. A rise in non-HSA-bound Zn^2+^ may also increase Zn^2+^ uptake/flux by endothelial cells, leukocytes, and platelets via zinc transporter proteins [32,48,89]. For platelets, this small elevation in free Zn^2+^ may be enough to sensitize them to other agonists and cause inappropriate activation [48]. Additionally, in the event of reduced Zn^2+^ binding by HSA, it is likely this Zn^2+^ binds other plasma proteins including those that mediate thrombosis in a Zn^2+^-dependent manner. 

There are several coagulation-related, Zn^2+^-binding plasma proteins that may bind an increased proportion of Zn^2+^ when HSA-Zn^2+^ interactions are compromised by NEFA binding. These include plasminogen, plasmin, tPA, fibrinogen, factor XIII, and histidine-rich glycoprotein (HRG) [31,38,63,90]. The last three proteins, in combination with HSA, are all constituents of α-granules and their local plasma levels transiently increase locally at the surface of activated platelets [38,91]. HRG is particularly relevant as Zn^2+^ modulates its affinity toward other coagulation proteins, impacting their functioning. HRG is relatively abundant in human plasma (*ca.* 1.5 μM), although this value is often elevated in cardiovascular disease [92,93]. HRG is well suited to binding divalent metal ions owing to its histidine-rich region, which possesses 10 binding sites for Zn^2+^ (*K_D_* = 1.63 × 10^5^) [94]. These considerations make it a likely candidate protein to bind Zn^2+^ prevented from binding to HSA by elevated NEFAs. Furthermore, modelling of Zn^2+^ speciation has supported the hypothesis that HRG can pick up HSA-free Zn^2+^ [94]. Finally, both these *a priori* and modelling assumptions are supported by the Coverdale et al. study, which found greater levels of Zn^2+^ present in HRG-containing plasma fractions upon addition of myristate [31]. HRG has several important binding partners that regulate homeostatic processes, such as activated Factor XIIa, plasminogen, fibrinogen, fibrin, and heparin. HRG can regulate coagulation through these interactions and, importantly, these interactions are enhanced in the presence of Zn^2+^ [95,96,97]. HRG can counter the anticoagulant effects of heparin, upon binding, by reducing the availability of heparin for antithrombin, thereby enabling thrombin activity to continue [95]. Interestingly, heparin neutralization can also be achieved through its complexation with fibrinogen and, notably, this interaction is also enhanced in the presence of Zn^2+^. Fibrinogen binds Zn^2+^ with a *K*_D_ of 9 μM and it, therefore, may represent another possible Zn^2+^-binding partner when plasma NEFA levels are elevated [98]. Thus, reduced Zn^2+^ buffering may also enhance thrombotic risk in the obese and diabetic states through enhanced complexation of HRG-heparin and/or fibrinogen-heparin with resulting impairment to heparin-mediated anti-coagulation [32].

To fully understand the impact of NEFAs on Zn^2+^-dependent coagulation, further work is required to fully elucidate changes in plasma Zn^2+^ speciation in the presence of elevated NEFAs’ concentrations. Some approaches have been developed to examine such “speciomic” changes in metals in complex systems such as plasma. Laser ablation-inductively coupled plasma mass spectrometry in combination with 2D-polyacrylamide gel electrophoresis can be used to analyze the proportion of Zn^2+^ associated with specific proteins following separation on a gel. This approach has already been successfully employed to examine the association of different metals with metalloproteins in plasma [99]. Similarly, proteins bound to individual metals can be identified through chromatographic fractionation (under near-native conditions) using gel filtration and/or ion-exchange chromatography. The individual proteins in fractions can be subsequently identified by gel electrophoresis/mass spectrometry and metal concentrations within fractions by inductively coupled plasma mass spectrometry, with individual metal–protein interactions inferred from principal component analyses of the resulting data [100]. Such methods may be employed to identify plasma coagulation proteins that bind a higher proportion of Zn^2+^ in the presence of high concentrations of NEFAs.

## 5. Evidence for the Zn^2+^-NEFA Switch as a Thrombotic Mechanism

There is indirect evidence available that suggests altered Zn^2+^ dynamics may play a role in thrombotic disease. Such evidence includes the observation that analbuminemia (HSA deficiency) is associated with hypercoagulability [101]. While this is likely to be a result of a combination of factors, including altered concentrations of coagulation proteins, it is apparent that this would impact plasma Zn^2+^ availability (potentially increasing the proportion of Zn^2+^ able to bind coagulation proteins) and, thus, Zn^2+^-dependent hemostasis. In the opposite manner, it can be assumed that conditions that would potentially decrease the availability of Zn^2+^ for coagulation proteins should beneficially impact cardiovascular health. It is noted that many effective medications for diabetes have chelating properties [102,103,104,105] and, thus, it is conceivable that chelation of Zn^2+^ from prothrombotic proteins by these agents contributes in their ability to lower cardiovascular risk. Additional support for this assumption may be provided by recent findings from the trial to assess chelation therapy (TACT). Chelation therapy involves the intravenous or oral administration of chelating agents to remove metal ions from the blood. The use of chelation therapy remains controversial and, up until 2002, no large-scale clinical trial had existed that could independently ascertain whether the practice impacted favorably on cardiovascular risk in certain groups [106]. TACT was employed to study the safety and efficacy of EDTA-based chelation in a post-myocardial infarction (MI) population. Treatment involved 40 weekly infusions with either the active chelating agent or a saline solution [107]. Overall, participants receiving the chelation infusion had an 18% reduced risk of reaching the primary endpoint (a composite of death from any cause, myocardial infarction, stroke, coronary revascularization, and hospitalization for angina) compared to those receiving the placebo infusion (*p* = 0.035) [108]. Approximately one-third of the patients studied in TACT had T2DM and, intriguingly, this subgroup was shown to derive the most benefit from the EDTA-based infusions. Indeed, the risk of the combined primary endpoint was reduced by 41% in T2DM patients receiving EDTA infusion compared to those receiving the placebo (*p* < 0.001). There was additionally a 52% relative reduction in the risk of recurrent MI (*p* = 0.015) and a 43% relative reduction in the risk of death from any cause (*p* = 0.011) [109]. This reported benefit may conceivably occur, at least in part, through chelation of non-HSA-bound Zn^2+^ and prevention of its binding to clot-forming proteins.

Several pieces of evidence suggest that NEFA-mediated alterations in Zn^2+^ speciation represent a potential contributory mechanism for thrombosis in obesity and T2DM. Our recent work suggests NEFA and Zn^2+^ can act synergistically in homeostatic processes relating to platelet aggregation, fibrin clotting, and fibrinolysis [88]. The evidence of this synergism has come largely from ex vivo and in vitro approaches. Indeed, we recently addressed the question of whether NEFAs alter fibrin clot formation and lysis in a Zn^2+^-dependent manner using turbidimetric studies that utilized either purified proteins (fibrinogen and HSA) or plasma, with clotting initiated by the addition of thrombin in both systems. Irrespective of the system studied, the maximum absorbance (indicative of the size and density of the clot formed) increased with Zn^2+^ at concentrations of 20–100 µM, with the addition of myristate having a synergistic effect on this parameter [88]. Moreover, turbidimetric analysis of plasma samples taken from age- and sex-matched groups of individuals with T2DM and controls (without diabetes) was performed, and the plasma concentrations of major NEFA species in the samples were measured using GC-MS. Clot density was significantly higher in the diabetic cohort, which also had significantly higher plasma NEFA concentrations. The positive association between clot density and NEFA concentration thus mirrored the effect of NEFAs on clot parameters observed in turbidimetric assays. The NEFAs myristate, palmitate, linolenate (18:3), oleate (18:1c9), cis-vaccenate (18:1c11), stearate, eicosapentaenoate (20:5), and arachidonate (20:4) were elevated in the T2DM group. The concentrations of myristate, palmitate, oleate, *cis*-vaccenate, and stearate positively correlated with maximum absorbance, supporting the concept that elevated NEFA levels contribute to increased thrombotic risk in T2DM, potentially through mishandling of plasma Zn^2+^. In the same study, the effects of Zn^2+^ and NEFAs on platelet aggregation were examined in vitro. The presence of myristate (but not octanoate, which does not significantly affect Zn^2+^-binding to HSA) increased maximum aggregation in a concentration-dependent manner (addition of 4 mol. eq. myristate significantly increased maximum aggregation in platelets-in-plasma) and this effect was potentiated by the addition of (100 μM) Zn^2+^. Most crucially, addition of the metal chelator *N*,*N*,*N*′,*N*′-tetrakis(2-pyridinylmethyl)-1,2-ethanediamine (TPEN) abolished not only the effect of Zn^2+^ but also that of the NEFA. This provides strong evidence that the effect of NEFA on platelet aggregation was mediated by its ability to displace Zn^2+^ from HSA. Thus, it appears likely that the allosteric mechanism that mediates cross-talk between Zn^2+^ and NEFA binding first identified in simple model systems operates in real plasma [94,110], that NEFAs induce changes in Zn^2+^ speciation [31], and that these changes drive prothrombotic events in obesity and T2DM [88].

## 6. NEFAs a Target for Therapy

Reduced Zn^2+^ buffering by NEFAs represents a likely pathophysiological mechanism linking elevated plasma NEFA levels to increased risk of thrombotic complications in obesity and T2DM. Thus, lowering plasma NEFA concentrations in these groups is likely to be of great clinical usefulness. Removal of excess adiposity via caloric restriction and physical activity is an established method of improving cardiovascular health among obese and diabetic obese individuals [111]. Appropriate exercise and nutrition impact favorably on cardiovascular risk markers including lipid profile and, importantly, a diet- and exercise-mediated return to metabolic normality is predicted to lower plasma NEFA levels. Bariatric surgery in obese individuals also leads to marked decreases in plasma NEFAs after 6 months [112,113,114,115]. In addition to these strategies, it may be possible to leverage the pleiotropic actions of current diabetes medications, given that treatment with these agents also has the potential to lower plasma NEFA concentrations.

One aspect of T2DM treatment involves the pharmacological management of diabetic dyslipidemia in which lipid-lowering drugs including statins and fibrates are commonly employed [116]. The ability of these agents to lower the incidence of cardiovascular events is supported by numerous randomized clinical outcome studies [117]. Their clinical benefit has been mainly attributed to their ability to impact on total plasma cholesterol (TC), low-density lipoprotein (LDL), and high-density lipoprotein (HDL) concentrations. However, it is apparent that these drugs also influence plasma NEFA levels and thus it is possible that they could be used to “normalize” elevated NEFA concentrations in obesity and T2DM. Statins are the most employed agents for the treatment of diabetic dyslipidemia. These drugs decrease plasma cholesterol levels by competitively inhibiting hydroxymethylglutaryl-CoA reductase, the principal rate-limiting enzyme in cholesterol biosynthesis. Statins also lower plasma cholesterol levels indirectly by increasing the expression of LDL receptors on cell surfaces, resulting in greater LDL uptake. Additionally, a recent meta-analysis has shown that these agents can significantly lower total plasma NEFA levels in individuals with T2DM, metabolic syndrome, or dyslipidemia [118]. Fibric acid derivatives or fibrates are employed secondarily to statin therapy. These agents act primarily by activating the peroxisome proliferator-activated receptor-alpha, (PPAR-α) which leads to an increase in NEFA oxidation and decreased triglyceride synthesis in the liver [119]. Several studies have investigated the effect of fibrate treatment on total plasma NEFA levels in different populations, which included individuals with various metabolic derangements (healthy subjects and subjects with T2DM, hypolipoproteinemia, hyperinsulinemia, hypertriglyceridemia, glucose intolerance, or metabolic syndrome) [120,121,122,123,124,125,126,127,128,129,130,131,132,133,134]. In all studies, plasma NEFA levels were found to be either unchanged or reduced except for a single study [128], in which fibrate treatment increased NEFA levels in individuals with hypertriglyceridemia and glucose intolerance.

Normalizing blood glucose concentrations represents another goal in the management of diabetes. This aspect of management involves the use of antihyperglycemic drugs such as metformin, sodium-glucose cotransporter 2 (SGLT) inhibitors, and glucagon-like peptide 1 (GLP-1) receptor agonists. Metformin is often the first choice of glucose-lowering drugs for T2DM patients. The drug improves insulin sensitivity by inhibiting gluconeogenesis in the liver [135]. Most studies, however, do not recognize any effect of metformin on plasma NEFA concentrations [136,137,138,139] and, thus, this agent is an unsuitable candidate for lowering NEFA levels in diabetic and obese diabetic patients. Interestingly, the ability of metformin to confer true cardiovascular benefit has come into question in recent years [140,141]. Meta-analyses have shown metformin was unable to confer significant benefit to all-cause mortality, cardiovascular mortality, MI, peripheral vascular disease, or stroke in T2D patients. GLP-1 receptor agonists and SGLT-2 inhibitors are two new classes of antihyperglycemic drugs that are both very effective at lowering blood glucose levels [142]. Additionally, these drugs also have numerous pleiotropic actions that may endow them with NEFA-lowering properties [143,144]. Glucagon-like peptide 1 (GLP-1) receptor agonists stimulate release of insulin from the pancreas in response to oral and intravenous glucose [145]. The GLP-1 agonist liraglutide has been demonstrated to be superior to placebo in reducing the primary composite of time to death, non-fatal MI, and non-fatal stroke in high-risk cardiovascular patients [146]. Two recent meta-analyses have additionally demonstrated that liraglutide beneficially influences lipid status in T2DM patients, causing significant reductions in TC, LDL, triglycerides (TG), and NEFAs [147,148]. Like GLP-1 receptor agonists, SGLT-2 inhibitors help to regulate glycemic control. These agents stimulate glycosuria (the excretion of glucose into the urine) by inhibiting SGLT-2-mediated glucose reabsorption in the proximal tubule of the kidneys [145,149]. T2DM patients treated with the SGLT-2 inhibitor empagliflozin have a reduced risk of the primary composite outcome of cardiovascular disease-related death, non-fatal MI, and stroke compared to T2DM patients receiving placebo [150]. Importantly, SGLT-2 inhibitors are predicted to lower plasma NEFA levels, given their ability to activate PPAR-α and, thus, promote fatty acid oxidation in a similar manner to fibrates [151].

**Figure 3:**
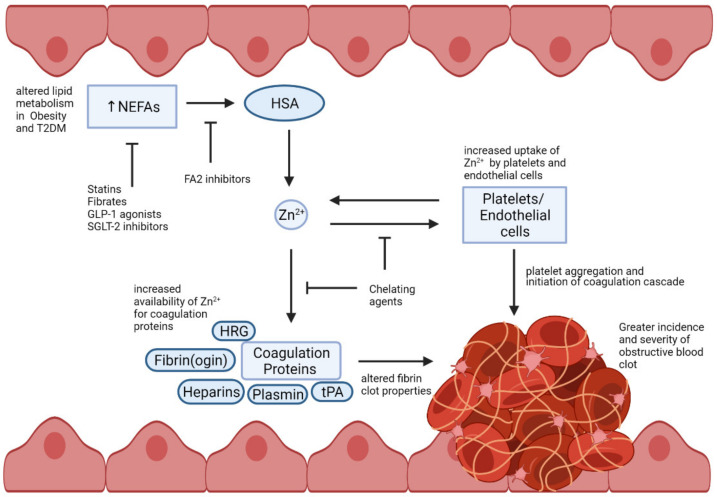
Potential Zn^2+^-mediated thrombotic routes in obesity and T2DM and targets for therapy. Altered lipid metabolism in obesity and T2DM results in elevated NEFA levels in the blood. NEFA binding at the FA2 site of HSA prevents procoagulant Zn^2+^ ions from binding at site A. The Zn^2+^ unable to bind HSA is redistributed among the cellular and protein components of the homeostatic mechanism, resulting in thrombotic events. This mechanism of thrombosis can be targeted by using lipid- and glucose-lowering drugs, which lower plasma NEFA levels. Therapeutics with chelating properties may also have potential to prevent free Zn^2+^ ions interacting with homeostatic components. FA2 inhibitors could also be developed to restore Zn^2+^ buffering by HSA. Created with BioRender.com (accessed on 13 September 2021).

Alternatively, it is noted that this prothrombotic mechanism could be targeted more specifically by employing inhibitors that selectively bind to the FA2 site of HSA in a manner that prevents NEFA binding but does not disrupt Zn^2+^ binding to site A. These inhibitors would, thus, protect the Zn^2+^-binding capacity of HSA at high NEFA concentrations, while still allowing the protein to carry NEFAs via its other binding sites. Importantly, prior to any potential therapeutic use, these molecules should first be used to assess the degree to which the NEFA-Zn^2+^ switch contributes to the prothrombotic state in obesity and T2DM. Indeed, to clarify the relevance of this mechanism, inhibitors directed against the FA2 site could be tested using coagulation assays in animal models. In particular, the potential protective effects of these molecules on fibrin clot formation/lysis and platelet function in the presence of different Zn^2+^ and NEFA concentrations should be ascertained. A summary of the mechanisms by which NEFAs impact on HSA-bound Zn^2+^ and how these may be targeted are shown in Figure 3.

## 7. Conclusions

Current evidence indicates that plasma NEFAs impact Zn^2+^ speciation via an allosteric mechanism (occurring at the FA2 site) on HSA in obese and T2DM disease states. In addition to the established mechanisms of NEFA-induced thrombogenesis, this dynamic is likely to contribute to thrombotic complications observed in these diseases. Further work is needed to identify the impact of certain NEFAs on Zn^2+^-HSA binding, particularly unsaturated NEFAs that have not yet been investigated in this context. In addition, mixtures of NEFAs that represent in vivo concentrations (in health and disease) should be investigated. Zn^2+^ is an important regulator of hemostasis and acts through multiple mechanisms to control coagulation. Knowledge of specific pathways through which it can act has come from in vitro and ex vivo studies. It is unclear which specific proteins or pathways may be activated by non-HSA-bound Zn^2+^ in vivo. However, methods exist to explore Zn^2+^ speciation in relevant systems to better understand which are of the most significance in thrombotic disease. Specific control of plasma NEFA levels, Zn^2+^ availability, or, indeed, Zn^2+^-dependent coagulatory processes are largely overlooked as pharmacotherapeutic strategies to limit cardiovascular events in at-risk populations. While appropriate nutrition and exercise are potential approaches to lower plasma NEFAs, a future strategy may be to exploit the pleiotropic effects of currently marketed drugs for the treatment of obesity and T2DM, given that many of these agents show NEFA-lowering properties outside of their primary mechanism of action. Additionally, when designing new obesity and T2DM therapeutics, greater focus should be placed on the ability of these drugs to control NEFA levels in addition to more established risk markers such as cholesterol, HDL, and LDL. Insights from TACT have revealed a potential benefit of chelation to cardiovascular health in high-risk groups and, therefore, future therapies for obesity and T2DM could also make use of compounds with chelating properties. These agents may exert their beneficial effect by blocking non-HSA-bound Zn^2+^ from interacting with components of hemostasis. Finally, more specific strategies that selectively target NEFA binding to FA2 or Zn^2+^-mediated interactions between macromolecules can potentially be developed. Such agents could be useful for the treatment or management of thrombotic complications and be employed as tools in studies designed to assess the degree to which NEFA-mediated alterations in plasma Zn^2+^ dynamics contribute to thrombotic disorders in high-risk groups.
